# Supplementary material for: Cromileptes altivelis microRNA Transcriptome Analysis upon Nervous Necrosis Virus (NNV) Infection and the Effect of cal-miR-155 on Cells Apoptosis and Virus Replication
Source: Viruses. 2022 Oct 3;14(10):2184. doi: 10.3390/v14102184 (PMC9609685; doi:10.3390/v14102184)

Supplement:

Table S1

Overview of the sequencing raw data

Sample	Reads count	Base count	Average length (bp)	Q10	Q20	Q30	GC percentage
3S1	12315159	923636925	75	99.99%	87.17%	84.11%	49.52%
3S2	10135519	760163925	75	99.99%	97.23%	95.78%	49.80%
3S3	12477154	935786550	75	99.99%	95.63%	93.41%	49.83%
**Average**	**11642611**	**873195800**	**75**	**99.99%**	**93.34%**	**91.10%**	**49.72%**
8S1	8787473	659060475	75	99.99%	97.19%	95.57%	51.04%
8S2	7660843	574563225	75	99.99%	97.11%	95.48%	51.13%
8S3	12130288	909771600	75	99.99%	95.14%	92.81%	49.54%
**Average**	**9526201**	**714465100**	**75**	**99.99%**	**96.48%**	**94.62%**	**50.57%**
PS1	10764356	807326700	75	99.99%	97.23%	95.59%	51.33%
PS2	11444615	858346125	75	99.99%	97.15%	95.60%	48.47%
PS3	12104485	907836375	75	99.99%	95.53%	93.15%	50.89%
**Average**	**11437819**	**857836400**	**75**	**99.99%**	**96.64%**	**94.78%**	**50.23%**

Table S2

Overview of the sequencing clean data

Sample	Reads count	Unique reads count	Bases count	Average length (bp)	Q10	Q20	Q30	GC percentage
3S1	10744450	372573	238819645	22.23	100.00%	97.82%	96.75%	42.51%
3S2	7973040	364965	179348243	22.49	100.00%	98.58%	97.91%	43.40%
3S3	10059217	572012	227486237	22.61	100.00%	97.83%	96.75%	43.33%
**Average**	**9592236**	**436517**	**215218042**	**22.44**	**100.00%**	**98.08%**	**97.14%**	**43.08%**
8S1	7517676	263931	167734058	22.31	100.00%	98.58%	97.93%	43.15%
8S2	6173908	281487	138670324	22.46	100.00%	98.51%	97.82%	43.54%
8S3	9890625	355980	219042501	22.15	100.00%	97.68%	96.53%	42.59%
**Average**	**7860736**	**300466**	**175148961**	**22.31**	**100.00%**	**98.26%**	**97.43%**	**43.09%**
PS1	9261268	389174	209944197	22.67	100.00%	98.57%	97.92%	44.08%
PS2	10476853	324004	235331173	22.46	100.00%	98.60%	97.97%	43.35%
PS3	10688703	283539	236058854	22.08	100.00%	97.80%	96.71%	42.50%
**Average**	**10142275**	**332239**	**227111408**	**22.40**	**100.00%**	**98.32%**	**97.53%**	**43.31%**

Table S3

The primers used for validation of miRNAs by qRT-PCR

miRNA name	Sequence	RT-pimer(5’-3’)	qPCR Forward primer
miR-132-3p	UAACAGUCUACAGCCAUGGUCG	GTCGTATCCAGTGCAGGGTCCGAGGTATTCGCACTGGATACGACCGACCA	GCGCGTAACAGTCTACAGCCA
miR-146b	UGAGAACUGAAUUCCAAGGGUG	GTCGTATCCAGTGCAGGGTCCGAGGTATTCGCACTGGATACGACCACCCT	CGCGTGAGAACTGAATTCCA
miR-155	UUAAUGCUAAUCGUGAUAGGGG	GTCGTATCCAGTGCAGGGTCCGAGGTATTCGCACTGGATACGACACCCCT	CGCGTTAATGCTAATCGTGAT
miR-194a	UGUAACAGCAACUCCAUGUGG	GTCGTATCCAGTGCAGGGTCCGAGGTATTCGCACTGGATACGACCCACAT	GCGCGTGTAACAGCAACTCC
miR-203b-5p	AGUGGUUCUCAACAGUUCAACA	GTCGTATCCAGTGCAGGGTCCGAGGTATTCGCACTGGATACGACCAAGTG	CGCGGTGAAATGTTTAGGAC
novel-110	AAUGUAGUAGACUUAAAGUAUA	GTCGTATCCAGTGCAGGGTCCGAGGTATTCGCACTGGATACGACTATACT	CGCGCGAATGTAGTAGACTTAA
novel-14-star	CUCACUGAACAAUGAGUGCAAC	GTCGTATCCAGTGCAGGGTCCGAGGTATTCGCACTGGATACGACGTTGCA	CGCGCTCACTGAACAATGAG
novel-144	UCAAGUAGAAUCGAAACUCCGU	GTCGTATCCAGTGCAGGGTCCGAGGTATTCGCACTGGATACGACACGGAG	GCGCGTCAAGTAGAATCGAAA
novel-221	UGAAGUCUGUGAUCUUGCAUCA	GTCGTATCCAGTGCAGGGTCCGAGGTATTCGCACTGGATACGACTGATGC	GCGCGTGAAGTCTGTGATCTT
novel-229	GUGAUUGACAUGACUAACA	GTCGTATCCAGTGCAGGGTCCGAGGTATTCGCACTGGATACGACTGTTAG	GCGCGGTGATTGACATGA
novel-41-star	UCGCCGGUCGAUGAAUGACA	GTCGTATCCAGTGCAGGGTCCGAGGTATTCGCACTGGATACGACTGTCAT	CGTCGCCGGTCGATGA
novel-441	UCUACGCUGUAACCUAUGCUGU	GTCGTATCCAGTGCAGGGTCCGAGGTATTCGCACTGGATACGACACAGCA	GCGCGTCTACGCTGTAACCTA
novel-607	CCGUUUCCACUGUAGAAGUUCCU	GTCGTATCCAGTGCAGGGTCCGAGGTATTCGCACTGGATACGACAGGAAC	GCGCCGTTTCCACTGTAGAA
novel-8-star	CGCUGGACAGGUUUGGGGGCGGC	GTCGTATCCAGTGCAGGGTCCGAGGTATTCGCACTGGATACGACGCCGCC	GCGCTGGACAGGTTTGGG

Table S4

PCR primer sequence information in this study.

Primer name	Sequence(5'-3')
Ca-U6 F1	ATTGGAACGATACAGAGAAGATT
Ca-U6 R1	GGAACGCTCCACGAGTTTG
mQ Primer R	AGTGCAGGGTCCGAGGTATT
cal-miR-155 mimic	UUAAUGCUAAUCGUGAUAGGGG (sense);<br>CCCCUAUCACGAUUAGCAUUAA (antisense)
cal-miR-155 inhibitor	CCCCUAUCACGAUUAGCAUUAA
cal-miR-155 mimics NC	UUGUACUACACAAAAGUACUG (sense);<br>GUACUUUUGUGUAGUACAAUU (antisense)
cal-miR-155 inhibitor NC	CAGUACUUUUGUGUAGUACAA
EF-α-RT-F	AGCCGACAGTGTAGAGGAGCAA
EF-α-RT-R	GCATGAACCAGGAGCCGTTGT
Cal-casp-3-RT-F	AGCCGACAGTGTAGAGGAGCAA
Cal-casp-3-RT-R	GCATGAACCAGGAGCCGTTGT
Cal-casp-6-RT-F	AGACAAGTGCAGGAGCCTCGTA
Cal-casp-6-RT-R	GCGTCCACCACCACCTCATTTG
Cal-casp-8-RT-F	TGGACGATGATGCCTTGGTGGT
Cal-casp-8-RT-R	GGTGAAGGGCGAGGTCAGTTCT
Cal-FADD-RT-F	GCTGCTGAAGGAGTGGAGGAAG
Cal-FADD-RT-R	CAGGTTGAAGTCGCAGGCTCTC
Cal-p53-RT-F	GTGCTTTGAGGTGCGTGTCTGT
Cal-p53-RT-R	TTGGTGCCGTTCTGCGTCTTC
Cal-BCL-2-RT-F	GGGATGGGATGCCTTTGTGGAG
Cal-BCL-2-RT-R	CAAGTGCTGCCAGACCGAAGAC
Cal-Bax-RT-F	GGCTGGCCTGGAAGACACAATG
Cal-Bax-RT-R	GCCGACTGACACGAAGTGGAGA
Cal-IL-6-RT-F	GCTGCCCTTCCTCCAACTTCAG
Cal-IL-6-RT-R	GCCGCCGTAGTATCTGACCTCT
Cal-IL-1β-RT-F	TCTGGGCATCAAGGGCACACA
Cal-IL-1β-RT-R	AACCGCACCATGTCGCTGTTC
Cal-IFN-γ-RT-F	TCTCTGGCTGTCCGTCTGTCAG
Cal-IFN-γ-RT-R	TGGTGGTCAGTGGTTCTCTGGA

Figure S1. Reads length distributions of total (A) and unique (B) sequences of miRNAs from control, 3D, and 8D libraries.

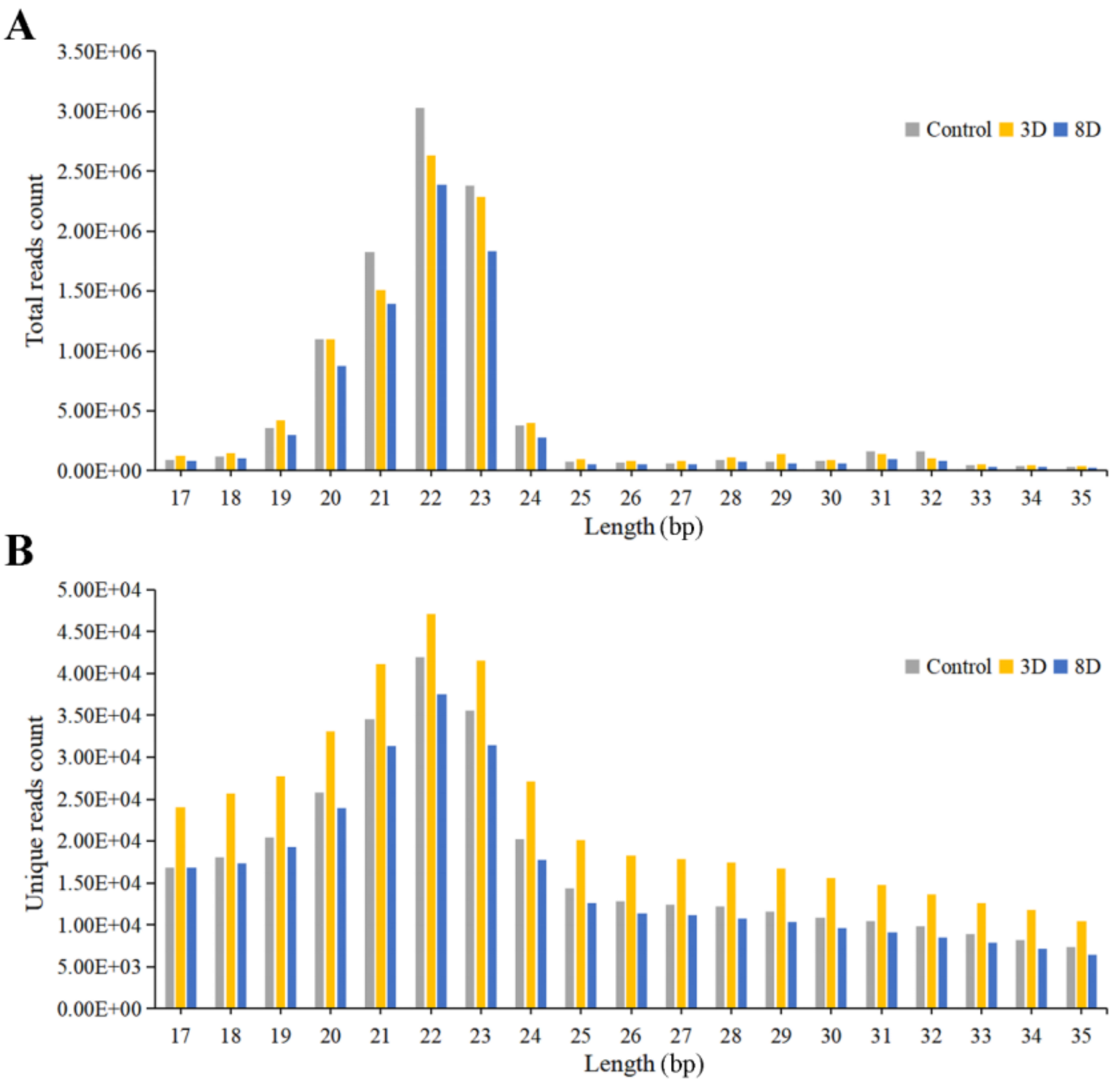

Figure S2. Annotation of small RNAs derived from sequencing of *C. altivelis* small RNAs libraries of 3D (A, B), 8D (C, D), and control (E, F) groups

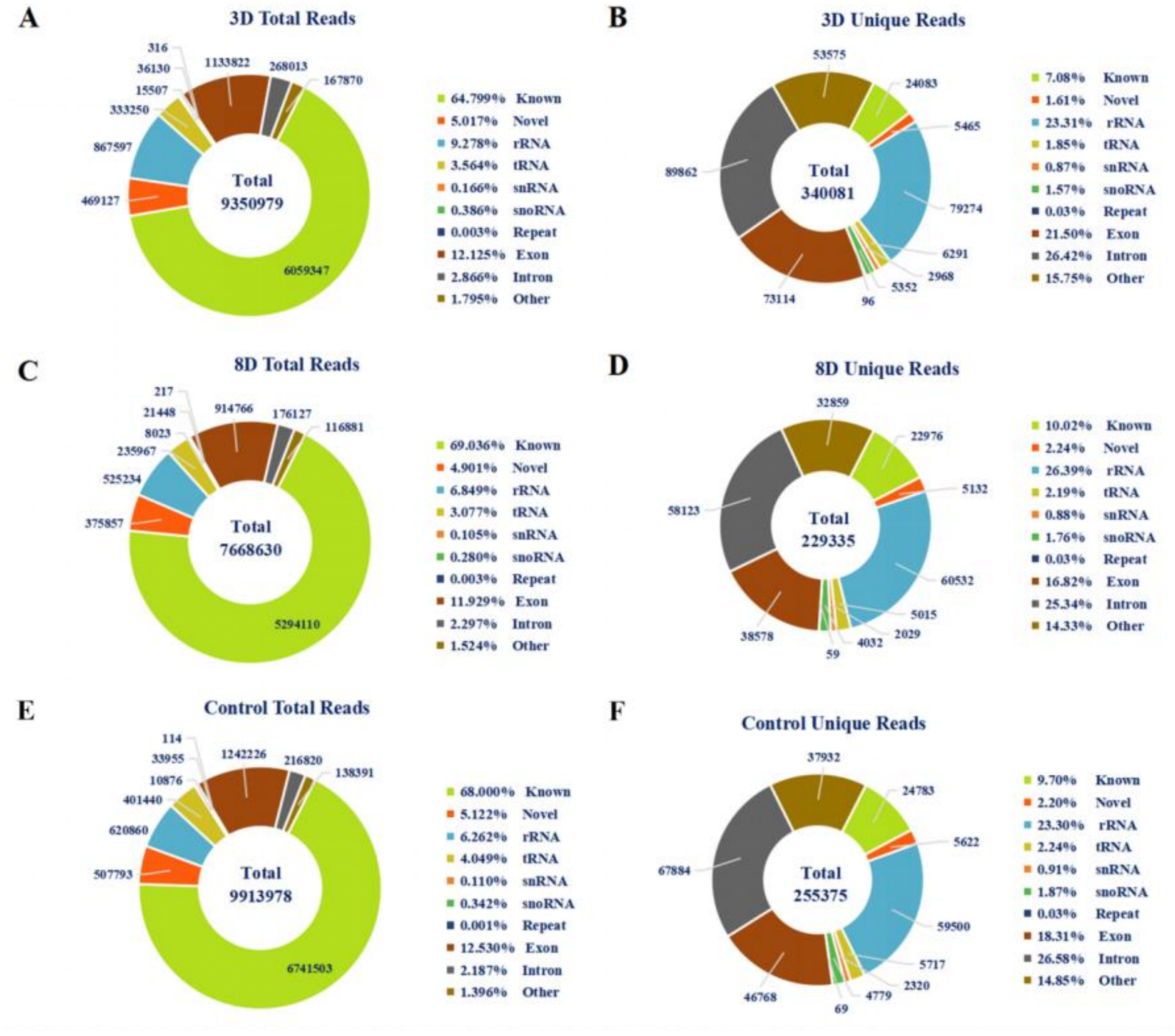

Figure S3. Revolutionary conservation analysis of *C. altivelis* miRNAs

(**A**) The miRNA clean reads were mapped onto 42 vertebrate species, covering the Mammalia, Aves, Reptilia, Amphibia, Osteichthyes, Cyclostomata and including 8 bony fish miRNAs from miRbase 22.1 using the miRDeep2 software (Version 2.0.0.8). (**B**) The miRNA families were analyzed to the animal kingdom after we sorted the 100 conserved miRNA families into nine groups base on the species evolution. miRNAs information of 25 species were downloaded from miRbase and their time-tree was built depend on the evolutionary timescale of life by TIMETREE 5 (http://timetree.org/). These species included two cephalochordates, eight fish, two amphibians, four mammals, two reptiles, two aveses, two uro-chordatas (uch), two non-chordatas (nch), one cyclostomata. A plus (+) or minus (−) symbol indicated a miRNA family existing or absence in the species on the left.

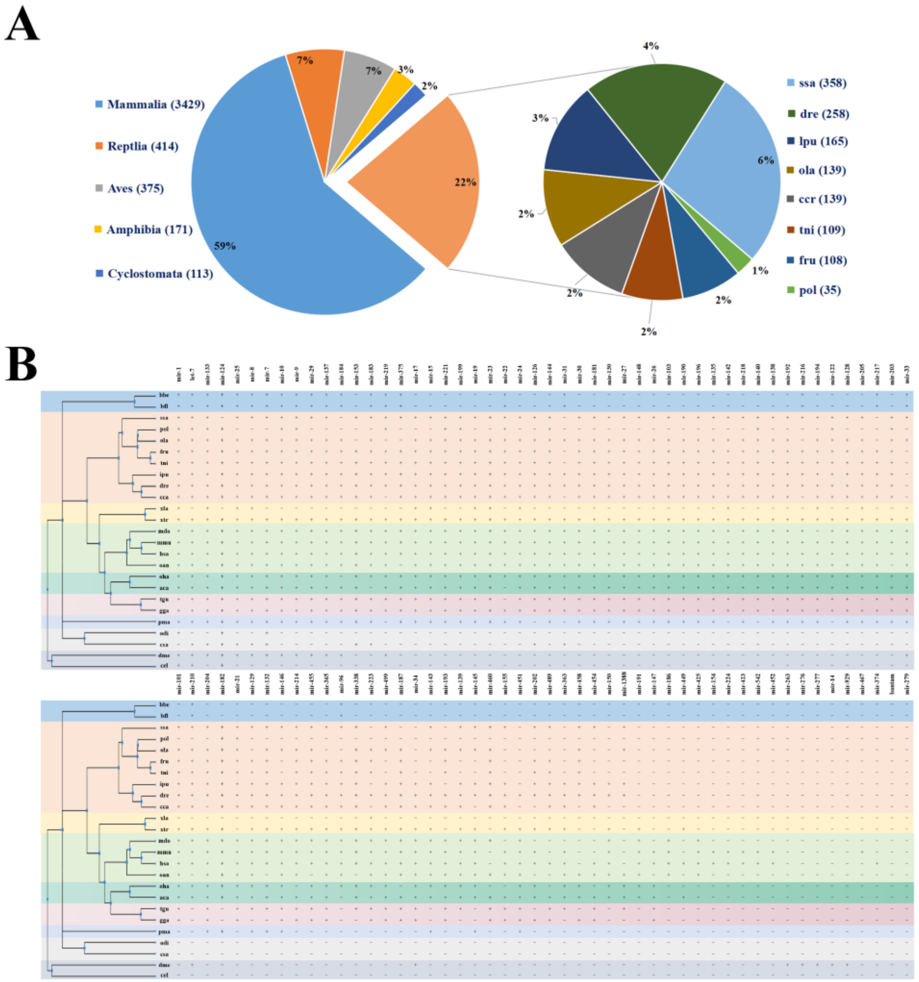

Supplement: Supplementary file 1 [file viruses-14-02184-s001.zip › viruses-1812104-supplementary.pdf]
